# Structural-equation-modelling of the tropism impact on achieving viral suppression within six months in naïve HIV patients

**DOI:** 10.7448/IAS.17.4.19679

**Published:** 2014-11-02

**Authors:** Carlo Mengoli, Samantha Andreis, Renzo Scaggiante, Mario Cruciani, Oliviero Bosco, Roberto Ferretto, D. Leone, Gaetano Maffongelli, Monica Basso, Loredana Sarmati, Massimo Andreoni, Giorgio Palù, Saverio Giuseppe Parisi

**Affiliations:** 1 Department of Molecular Medicine, University of Padova, Padova, Italy; 2 Center of Community & Medicine and HIV Outpatient Clinic, ULSS 20, Verona, Italy; 3 Clinical Infectious Diseases, Schio Hospital, Schio, Italy; 4 Clinical Infectious Diseases, Tor Vergata University, Rome, Italy

## Abstract

**Introduction:**

Aim of the study was to evaluate the relevance of baseline (BL) plasma tropism of HIV on the achievement of a viral suppression within six months of antiviral therapy (ARV) in naïve patients by a structural-equation-modelling.

**Materials and Methods:**

Two-hundred and twenty-seven patients were enrolled; viral tropism on plasma was determined at baseline (BL) by sequencing and interpretation by genotopheno algorithm. Booster atazanavir or lopinavir , or efavirenz or nevirapine were used, in combination with either abacavir/lamivudine or tenofovir-emtricitabine.

**Results:**

X4-tropism correlate negatively with CD4 cell count at BL and follow-up (FU), and CD4 correlate negatively with BL-plasma viremia (PLV). BL-PLV correlate positively with FU-PLV. We have developed the hypothesis that the variables BL-CD4 and BL-PLV represent a mediators chain among X4-tropism and outcome of plasma viraemia at six months. This model, after structural-equation-modelling (SEM, Stata13), is shown in Figure 1. The indirect effect of X4-tropism on Fup-PLV is significant (p<0.01) but about 10 fold lower than the direct effect by BL-PLV. X4-tropism also has a direct negative effect on BL-CD4 (p<0.001) and an indirect positive effect on BL-PLV (p<0.001), irrespective of the drug regimen. Path model explaining direct and mediated effects of “tro (tropism),” “gender,” “age,” “cd0 (BL-CD4)” and “lrna0 (BL-PLV)” on the final outcome (“lrna1-Fup-PLV),” where “tro,” “gender,” and “age” are exogenous, cd0 and lrna0 are endogenous (mediators). Numbers on the arrows indicate direct effects. Circles indicate residuals related to endogenous/dependent variables; numbers near to circles are the corresponding variances.

**Conclusions:**

This model shows the relevance of BL-tropism on the outcome of plasma viraemia in naïve patients after six months of therapy, irrespective of drug regimen used.

**Figure 1:**
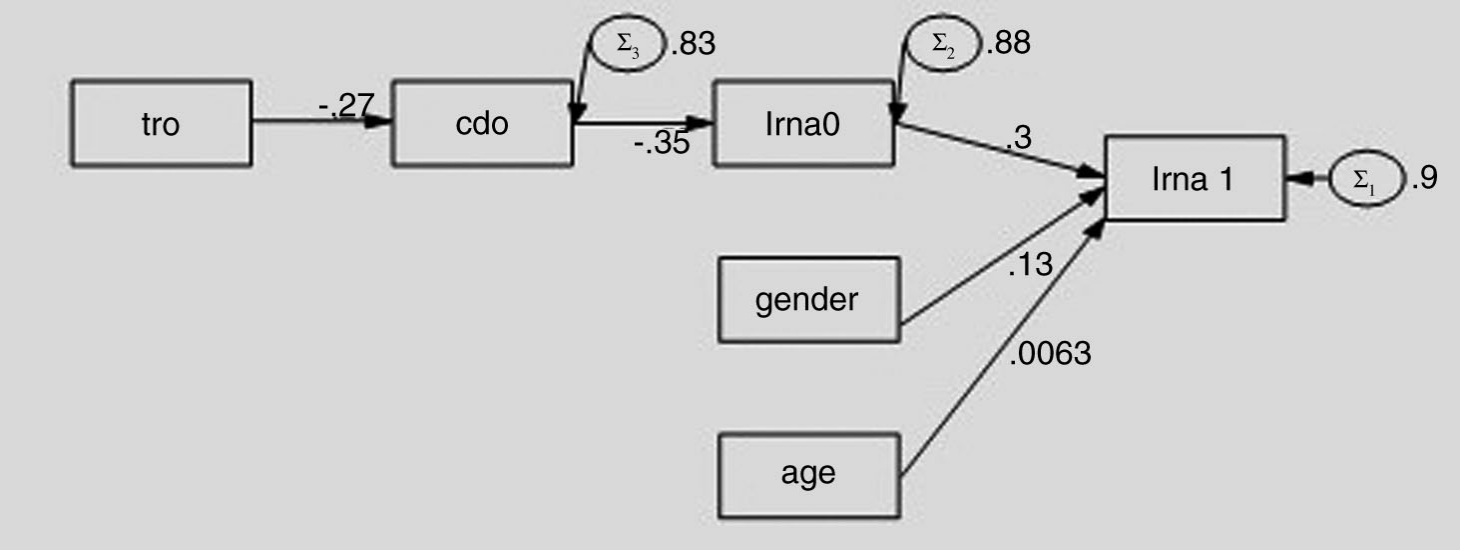
Path analysis model explaining direct and mediated effects of “tro”, “gender”, “age”, “cd0” and “lrna0” on the final outcome (lrna1, where tro, gender, and ageare exogenous, cd0 and lrna0 are endogenous (mediators. Numbers on the arrows indicate direct effects. Circles indicate residuals related to endogenous/dependent variables; numbers near to circles are the corresponding variances.

